# Effect of acupoint therapies on prostatitis: A systematic review protocol

**DOI:** 10.1097/MD.0000000000018967

**Published:** 2020-02-07

**Authors:** Kun Zhu, Yifeng Shen, Yi Zhu, Lan Li, Yaodong You

**Affiliations:** a Hospital of Chengdu University of Traditional Chinese Medicine; b Chengdu University of Traditional Chinese Medicine, PR China.

**Keywords:** acupoint therapies, prostatitis, protocol, systematic review

## Abstract

**Background:**

Prostatitis is a common urinary infectious disease. Acupoint therapies, including massage, acupuncture, acupoints injection, acupressure, and moxibustion, showed clinical and long-lasting benefits for prostatitis, but the efficiency of acupoint therapies was poorly evaluated. The current review is attempted to evaluate the efficacy and safety of the different acupoint-based therapies for prostatitis.

**Methods:**

Randomized controlled trials (RCTs) assessing acupoint therapies for prostatitis are included in the following databases: Web of Science, PubMed, Science Direct, Wan Fang Data Knowledge Service Platform, Chinese Biomedical Literature Database (CBM), Chinese Scientific Journal Database (VIP database), China National Knowledge Infrastructure (CNKI), and EMBASE. All RCTs on acupoint or related interventions will be included we will also conduct unpublished academic research data. Databases will be searched from inception to Dec 2019. Study inclusion, data extraction, and quality assessment will be performed independently by 2 reviewers. Assessment of risk of bias and data synthesis will be performed by RevMan 5.3 software. Cochrane criteria for risk-of-bias will be used to assess the methodological quality of the trials.

**Results:**

According to the data of effective rate, quality of life, the cure rate, the recurrent rate, and the complications rate, this study will provide an evidence-based review of acupoint therapies for prostatitis.

**Conclusion:**

This systematic review will evaluate the efficacy, and safety of the different acupoint-based therapies for prostatitis, we will recommend which acupoint therapies may be considered as the effective treatment for prostatitis, and show how it might work.

**PROSPERO registration number:**

DOI 10.17605/OSF.IO/H7KQJ.

## Abstract 2

Supplemental Digital Content is available in the text

## 1 Introduction

Prostatitis is a common urinary tract disease, which is generally accompanied by lower urinary tract symptoms. Except Benign Prostate Hyperplasia (BPH) and prostate cancer, prostatitis is the third commonly found urinary tract disease in males.^[1]^ It has been ranked fourth among the 20^[2]^ principal diagnostic diseases in the United States. Prostatitis is estimated to affect up to 30% of the males in their lifetime, which is especially prevalent in men aged between 35 and 50. The clinical features of prostatitis include perineal ache and penile tip pain in association with other urinary tract symptoms.^[3]^ Prostatitis, especially chronic abacterial prostatitis, called “chronic pelvic pain syndrome” (CPPS), frequent failure to be alleviated by medical treatment.^[4]^ Hence, effective management of prostatitis clinical symptoms is beneficial for patients’ life quality.

The present study shows prostatitis is subclassified into acute bacterial prostatitis (category I), chronic bacterial prostatitis (category II), CPPS (category III), and symptomatic prostatitis (category IV).^[1]^ Novel therapies include antidepressants, anti-inflammatory therapy, physiotherapy, alpha-blocker, neuroleptics, anti-anxiolytics, etc.^[5–7]^ However, because of its complex etiology and pathogenesis, complete control of prostatitis is difficult. With poor response to therapy, prostatitis presents negative adherence in improving symptoms (pain, voiding) and quality of life domain subscores. Besides, using anti-inflammatory drugs will lead to some side effects. According to related research,^[8]^ acupoint therapies of CP/CPPS is encouraging.

Acupoint therapies include massage, acupuncture, acupoints injection, acupressure, and moxibustion, which are safe medical procedures with minimal side effects for prostatitis’ clinical symptoms. Previous systematic review has shown that acupuncture-point stimulation would alleviate some symptoms of prostatitis such as perineal ache and penile tip pain in association with other urinary tract symptoms; however, these studies mainly showed the efficacy of single acupoint-based therapy for prostatitis, they have not been able to explain the treatment mechanism of different acupoint therapies and there have no reliable data to evaluate these traditional treatments scientifically and systematically. To evaluate the overall safety and effectiveness of acupoint therapies in prostatitis, we will undertake an evidence-based medicine systematic review, the validated outcome will give acupoint therapies recommendations for physicians.

## 2 Methods

### 2.1 Inclusion criteria for study selection

#### 2.1.1 Types of studies

All the randomized controlled trials (RCTs) of acupoint therapies for the management of prostatitis will be included. Writing language will be limited to Chinese and English. The quality of the studies should be restricted. We will exclude articles focus on case reports, non-RCTs, or RCT protocol.

#### 2.1.2 Types of patients

Study populations inclusion criteria will be all patients diagnosed with prostatitis and there are no restrictions on population ages, race, and education status.

#### 2.1.3 Types of interventions and controls

##### 2.1.3.1 Experimental interventions

The patients will be treated with acupoint therapies, including massage, acupuncture, acupoints injection, acupressure, and moxibustion. According to different types of prostatitis, the different internationally recognized therapy such as antimicrobial therapy, anti-inflammatories, urinary drainage will be received in the control group. The following comparison was included: firstly, acupoint therapies compared to drugs. Secondly, with the use of internationally recognized therapy and acupoint therapies together compared to the same internationally recognized therapy. Thirdly, acupoint therapies compared to sham/placebo acupoint therapies. The location of acupoints refers to the WHO standard acupuncture point locations in the Western Pacific Region.^[9]^ To meet the object of this review, we have no restriction on the type of selections of acupoints, therapies, materials, treatment time and methods, and course of treatment, and we will exclude the comparison between 2 different types of acupoint therapies or surgical procedures.

#### 2.1.4 Types of outcome measures

##### 2.1.4.1 Primary outcomes

The primary outcome measure consists of efficacy and expressed prostatic secretion (EPS) score.

##### 2.1.4.2 Secondary outcomes

The secondary outcomes are defined as changes in International prostatic symptom score (IPPS), the quality of life, recurrence rate, and side effects. Adverse events observed by acupuncturists from interventions will be collected as measurement.

### 2.2 Search methods for the identification of studies

#### 2.2.1 Data sources

RCTs assessing acupoint therapies for prostatitis are included in the following databases: Web of Science, PubMed, Science Direct, Wan Fang Data Knowledge Service Platform, Chinese Biomedical Literature Database (CBM), Chinese Scientific Journal Database (VIP database), China National Knowledge Infrastructure (CNKI), and EMBASE. All RCTs on acupoint therapies or acupoint-based related interventions will be included, we will also conduct unpublished academic research data. Databases will be searched from inception to Dec 2019. Two reviewers (KZ and YS) will search the data and assess the quality independently. The reference lists of review articles will be conducted and the following search terms will be used: acupuncture, acupressure, acupoint, acupoints injection, knead, reflexology, chiropra, massage, moxibustion, prostatitis, acute bacterial prostatitis, chronic prostatitis, chronic pelvic pain syndrome, asymptomatic inflammatory prostatitis. And we will take the search strategy which is obtainable in Supplemental Digital Content (Appendix A) for searching the database.

#### 2.2.2 Searching other resources

The authors will also search relevant trials from Clinical Trials.gov, Google Scholar, and WHO International Clinical Trials Registry Platform. The reference lists of the trials related to acupoint therapies on prostatitis will be considered.

### 2.3 Study selection

Researchers will import the literature retrieved to the Endnote X8 software and exclude the duplicate data. Literature search and literature screening will be performed independently by 2 reviewers referring to the inclusion criteria to exclude irrelevant studies. The researchers will read the full text of relevant articles to confirm the final inclusion of studies. For unclear dates, the researchers will contact the author for details to determine whether this literature would be included. Any disagreement between reviewers will be resolved by discussion or a third rater.

### 2.4 Risk of bias assessment

Two reviewers will evaluate the risk of bias for the RCTs included based on the Cochrane collaboration tool.^[10]^ Seven dimensions will be included: random sequence generation, allocation concealment, the blinding method for patients, researchers and outcomes assessors, incomplete outcome data, and selective reporting.^[11]^ Risks of bias will be divided into low, unclear, and high, respectively.^[12]^

### 2.5 Statistical collection and analysis

#### 2.5.1 Data extraction and management

Basic information will be extracted systematically and the researchers will apply the Jadad scale^[13]^ (3 questions on randomization, blinding, inclusion, and withdraws) to assess the quality of the report. The outcomes and methodological quality and hence bias risk will also be extracted. Data gathering will be done by 2 reviewers independently. And if they are inconsistent in the process, they will discuss the results. A third reviewer will be consulted to resolve the doubts. The researchers will contact corresponding authors for unclear details.

#### 2.5.2 Measurements of treatment effect

The continuous data will be analyzed by mean differences (MDs) with 95% confidence intervals (95% CIs). And standardized mean differences (SMD) analysis with 95% CIs will be included in the meta-analysis.

#### 2.5.3 Assessment of heterogeneity

The heterogeneity of research results will be assessed with the *χ*^2^ test and quantified with the *I*^2^ statistic test. If the *P*-value >.1 in the *χ*^2^ test and the *I*^2^ < 50%, the researchers will accept the model.

#### 2.5.4 Assessment of reporting biases

The funnel plot will be used to detect potential reported biases when the number of included trials >5. Egger's test^[14]^ and Begger's analysis can also assess the reporting bias.

#### 2.5.5 Data synthesis

The researchers will use Revman version 5 software to synthesize extracted data. If the results have no significant heterogeneity, the researchers will apply the fixed effect model to conduct a meta-analysis. The random effect model will be considered if the researchers find some degree of heterogeneity. If there is significant heterogeneity, we will take the subgroup analysis.

#### 2.5.6 Subgroup analysis

The researchers will perform a subgroup analysis if there is significant heterogeneity among the outcomes. The types of acupoint therapies will be considered firstly, secondly, we will perform the frequency of treatment. The types of disease, different chemotherapy drugs and the age, gender, and the patients’ race will also be included.

#### 2.5.7 Sensitivity analysis

A sensitivity analysis will be performed to check the robustness of the final results.

#### 2.5.8 Grading the quality of evidence

The researchers will take the Grading of Recommendations, Assessment, Development, and Evaluation (GRADE) to evaluate the quality of these outcomes. The outcomes will be divided into very low, low, moderate, or high.^[15]^

## 3 Discussion

With the advantage of less side effects and inexpensiveness, acupoint therapies are highly accepted. Some studies have shown that some acupoint-based therapies could alleviate some symptoms of prostatitis such as perineal ache and penile tip pain in association with other urinary tract symptoms.^[16–18]^ A report^[19]^ states that acupuncture (based on the acupoint therapies) has been practiced on a wide range of physiological disorders including pain, infection, neurological disorders, urogenital disorders. But these studies mainly showed the efficacy of single acupoint-based therapy for prostatitis, they have not been able to explain the treatment mechanism of different acupoint therapies and there have no reliable data to evaluate these traditional treatments scientifically and systematically.

**Figure 1:**
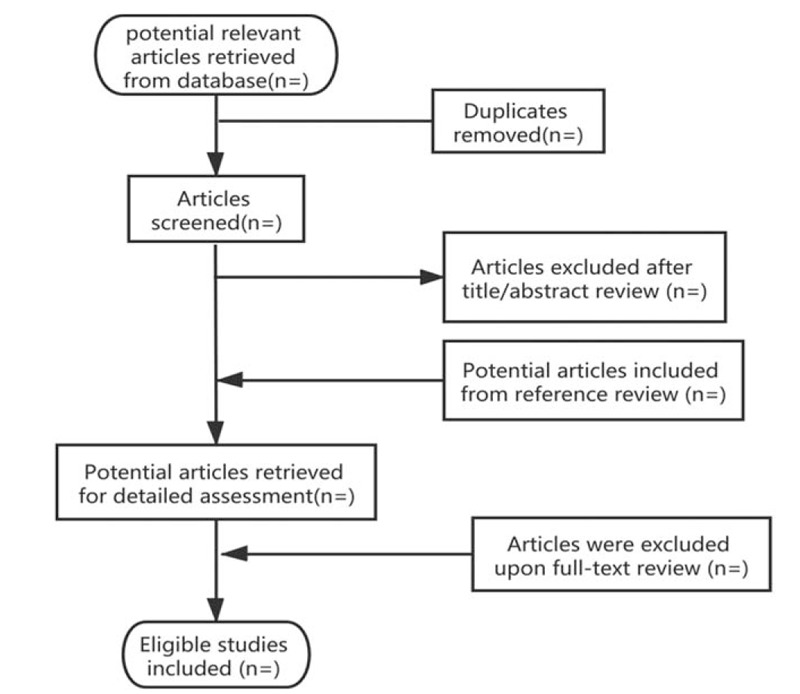
Flow diagram of studies identified.

This study will review currently researches, and this study aims to evaluate the efficacy and safety of the different acupoint-based therapies for prostatitis. The outcomes will recommend which acupoint therapies may be considered as effective treatment for prostatitis, and we will also show how it might work. The flow chart for this systematic review has been provided in Figure 1.

There might be some limitations of this review. Firstly, in this review, only studies published in English or Chinese will be included that might cause a potential risk of publication bias. Secondly, different types of acupoint therapies, selections of chemotherapeutics, and stage of prostatitis might run the risk of heterogeneity. Different measurements and tools might lead to inconsistent levels of outcomes.

## Author contributions

YY is the guarantor of the article. The manuscript was drafted by KZ and YS. LL and YZ developed the search strategy. YS and LL will independently screen the potential studies and extract data. KZ and YZ will assess the risk of bias and finish data synthesis. YY will arbitrate any disagreement and ensure that no errors occur during the review. All review authors critically reviewed, revised, and approved the subsequent and final version of the protocol.

## Supplementary Material

Supplemental Digital Content
