# Supplementary material for: Effect of acupoint therapies on prostatitis: A systematic review protocol
Source: Medicine (Baltimore). 2020 Feb 7;99(6):e18967. doi: 10.1097/MD.0000000000018967 (PMC7015636; doi:10.1097/MD.0000000000018967)
Supplement: Supplemental Digital Content [file medi-99-e18967-s001.doc]

**Supplemental Digital Content (Appendix A). Search strategy used in PubMed database**

1.“prostatitis"[Title/Abstract] OR “Acute Bacteria Prostatitis"[Title/Abstract] OR “Chronic Bacterial Prostatitis”[Title/Abstract] OR “prostatodynia” [Title/Abstract]

2."Acupoint"[Title/Abstract] OR "acupoint threapy"[Title/Abstract] OR "acupoint application"[Title/Abstract]

3. "Acupuncture"[Title/Abstract] OR "Acupressure"[Title/Abstract] OR "Massage"[Title/Abstract] OR "Moxibustion"[Title/Abstract] OR "acupoints injection"[Title/Abstract] OR "chiropra"[Title/Abstract] OR "reflexology"[Title/Abstract] OR "knead"[Title/Abstract] OR "[auricular point](http://www.baidu.com/link?url=lys06wnUGK__0J98JtWd8hr-307ToiaoPRWy2XbOIrULbdYczhRrXvM2E-PglsEoLQ27rFiJGmGue2BcAmTFfMV1i6v7U5evL3bbCgdVDOc1yqYw4AhtguoC9kATYzXx)"[Title/Abstract]

4.#2 OR #3

5. "randomized controlled trial"[Title/Abstract] OR "controlled clinical trial"[Title/Abstract]

6.#1 AND #4 AND #5
